# The impact of whole lung irradiation in lung metastatic rhabdomyosarcoma: A pooled analysis of two European trials and one European registry

**DOI:** 10.1002/cncr.70530

**Published:** 2026-07-23

**Authors:** Amadeus T. Heinz, Philip Heesen, Johannes H. M. Merks, Véronique Minard‐Colin, Martin Ebinger, Alison L. Cameron, Semi Harrabi, Henry Mandeville, Reineke A. Schoot, Jörg Fuchs, Gabriela Guillen, Ewa Koscielniak, Julia C. Chisholm, Monika Sparber‐Sauer, Gianni Bisogno

**Affiliations:** ^1^ Department of Pediatric Hematology and Oncology University Children’s Hospital Tuebingen Tuebingen Germany; ^2^ Stuttgart Cancer Center, Zentrum für Kinder‐, Jugend‐ und Frauenmedizin (Olgahospital) Pädiatrie 5 (Pädiatrische Onkologie, Hämatologie, Immunologie) Klinikum der Landeshauptstadt Stuttgart Stuttgart Germany; ^3^ University Hospital USZ University of Zurich Zurich Switzerland; ^4^ Princess Máxima Center for Pediatric Oncology Utrecht the Netherlands; ^5^ Division of Imaging and Oncology University Medical Center Utrecht University of Utrecht Utrecht the Netherlands; ^6^ Department of Pediatric and Adolescent Oncology Gustave Roussy Université Paris‐Saclay Villejuif France; ^7^ Bristol Haematology and Oncology Centre University Hospitals Bristol and Weston NHS Foundation Trust Bristol UK; ^8^ Department of Radiooncology and Radiotherapy Heidelberg University Hospital Heidelberg Germany; ^9^ Department of Radiotherapy Royal Marsden NHS Foundation Trust Institute of Cancer Research Sutton UK; ^10^ Department of Pediatric Surgery and Urology University Children’s Hospital Tuebingen Germany; ^11^ Pediatric Surgery Department Hospital Universitari Vall d'Hebron Universitat Autònoma de Barcelona Barcelona Spain; ^12^ Medical Faculty University Tuebingen Tuebingen Germany; ^13^ Children and Young People's Unit The Royal Marsden NHS Foundation Trust Institute of Cancer Research Sutton UK; ^14^ Department of Women’s and Children’s Health University of Padua Padua Italy; ^15^ Pediatric Hematology Oncology Division University Hospital of Padua Padua Italy

**Keywords:** CWS, EpSSG, lung irradiation, lung metastases, response, rhabdomyosarcoma, single site metastases, WLI

## Abstract

**Background:**

Lung metastases in patients with metastatic rhabdomyosarcoma (RMS) have not been treated uniformly across Europe. This provides comparison of the impact of whole lung irradiation (WLI).

**Methods:**

Lung‐metastatic patients included in the Cooperative Weichteilsarkom Studiengruppe‐IV 2002, European Pediatric Soft Tissue Sarcoma Study Group MTS 2008 or the Soft Tissue Sarcoma Registry received four‐ or six‐drug chemotherapy, surgery, and/or irradiation (radiotherapy) of the primary tumor. Treatment of lung lesions consisted of metastasectomy, WLI, or no local treatment according to protocols.

**Results:**

A total of 238 patients with lung‐metastatic RMS were included. Half of these patients (*n* = 119/238) had lung metastases only (median age, 6.6 years), mainly classified as embryonal RMS (*n* = 93/119, 78%). Lung‐only patients underwent metastasectomy (*n* = 17) and/or WLI (*n* = 31) or no local treatment of lung metastases (*n* = 71). Early complete response of lung metastases was associated with favorable 3‐year overall survival (*p* = .009). A trend toward improved event‐free survival after WLI could be identified in patients ≥10 years old (hazard ratio [HR], 2.7; 95% CI, 0.8–9.6), but not in patients under 10 years old (HR, 0.86; 95% CI, 0.44–1.66). Lung–relapse‐free survival (lung‐RFS) was not influenced by WLI or metastasectomy in the univariable and multivariable analyses. When analyzing all lung‐metastatic patients (including those with metastases in other sites, *n* = 238), WLI in patients older than 10 years was a significant prognostic factor (HR, 2.2; 95% CI, 1.0–4.9).

**Conclusions:**

The analysis failed to show a significant benefit of WLI in patients with lung‐metastatic RMS, apart from the subgroup of patients older than 10 years. Lung‐RFS was not influenced by WLI.

## Abstract 2

The results of two European trials and one registry were pooled together to analyze the potential benefit of whole lung irradiation in lung‐metastatic rhabdomyosarcoma patients. The analysis failed to show a significant benefit of whole lung irradiation in these patients, apart from the subgroup of patients older than 10 years.

## INTRODUCTION

The prognosis for children and adolescents with rhabdomyosarcoma (RMS) has progressively improved. With modern treatment protocols, more than 80% of patients with localized disease can now be cured.^1^ However, significantly less progress has been made for children presenting with metastatic disease at diagnosis whose survival rates remain poor. These patients represent approximately 15%–20% of the entire RMS population and often exhibit distant metastases in various organs, with the lungs being the most common site.^2^, ^3^, ^4^ Local control of lung metastases remains a critical challenge in improving cure rates for this group.

In North American protocols, whole lung irradiation (WLI) has been consistently recommended for patients with lung metastases.^3^, ^5^ In contrast, European protocols show greater variability: the European pediatric Soft Tissue Sarcoma Study Group (EpSSG) recommended WLI,^6^, ^7^, ^8^ whereas Cooperative Weichteilsarkom Studiengruppe (CWS) protocols did not include a clear recommendation.^9^, ^10^, ^11^ Despite these recommendations, the application of WLI has been inconsistent in both Europe and North America.^6^, ^12^, ^13^

To date, there is no clear evidence supporting these radiotherapy (RT) recommendations. No prospective randomized trial has evaluated the role of WLI and retrospective studies from the EpSSG, the Children's Oncology Group (COG), and CWS have reported conflicting outcomes. Some analyses suggest that WLI improves event‐free survival (EFS),^5^, ^7^, ^8^, ^12^, ^13^ whereas others found no significant difference in EFS between irradiated and nonirradiated patients.^9^, ^10^, ^11^, ^14^

Here, we present a pooled retrospective analysis of patients with lung‐metastatic RMS enrolled in EpSSG MTS 2008, CWS‐IV 2002, and the Soft Tissue Sarcoma Registry (SoTiSaR). The goal was to show the impact of WLI in a larger cohort of patients with lung metastases, independently of the specific protocol in which they were enrolled. The main analysis focuses on treatment and prognosis of patients with lung‐only metastatic RMS, followed by an additional analysis including patients with metastatic RMS of lung and other metastatic sites.

## MATERIALS AND METHODS

### Patients

EpSSG MTS 2008 (2009–2016, NCT 00379457),^8^, ^12^ CWS‐IV 2002 (2003–2010, including patients registered in CWS‐DOK‐IV‐2004),^11^ and SoTiSaR^14^, ^15^, ^16^ (2009–2019) included patients with a histopathologically proven diagnosis of RMS who were 0–21 years of age. Testing of fusion status^17^ was not standard of care during the time the trials were conducted.

All patients with RMS and lung metastases (excluding pleural lesions), with or without other metastatic sites, diagnosed between 2003–2019 with follow‐up until August 2023, were included in the current analysis with a median follow‐up of 7.5 years in surviving patients (interquartile range [IQR], 5.1–9.4 years).

### Definition of terms

The diagnostic work up included computed tomography (CT) and/or magnetic resonance imaging (MRI) of the primary tumor and locoregional lymph nodes. Metastatic disease was assessed by chest CT, optionally whole‐body MRI, technetium bone scan or positron emission tomography CT, cerebral MRI, and bone marrow aspiration/bone biopsy (two to four sites). Oberlin risk factors were defined according literature^2^ and included (1) age <1 year or ≥10 years, (2) primary tumor located in extremities, “other” sites or cancer of unknown primary, (3) metastases in three or more organ systems, and (4) evidence of bone or bone marrow metastases.

Response was assessed after three to four chemotherapy cycles (7–10 weeks) for the primary tumor (PT), lymph nodes, and metastases. Response of the primary tumor was assessed by 3D‐volumetric measurements and defined as complete response (CR) if there was complete disappearance of disease and partial response (PR) in patients with more than one‐third tumor volume reduction. Progressive disease (PD) was defined in CWS‐IV 2002^11^ and CWS Guidance^18^ as any increase in tumor volume of primary tumor or lymph nodes or appearance of new lesions in patients who had not achieved prior CR, in MTS 2008^8^ as an increase of tumor volume by 40% or appearance of new lesions. Stable disease (SD) was defined as any response not fulfilling the criteria for PR or PD. For lung metastases and/or other distant metastatic sites, response was classified as CR, non‐CR and PD.

### Treatment

All patients received multimodal treatment with chemotherapy and local therapy of primary tumor and regional lymph nodes in the form of surgery and/or radiotherapy. Intensive chemotherapy in MTS 2008 comprised four cycles of ifosfamide, vincristine, actinomycin‐D, and doxorubicin (IVADo) followed by five cycles of ifosfamide, vincristine, and actinomycin‐D.^8^ This was followed by maintenance chemotherapy, consisting of 12 cycles of daily low‐dose cyclophosphamide (CYC) for 4 weeks and weekly vinorelbine (CYC/VNB) administered in 3 out of 4 weeks. In CWS‐2002‐IV, intensive chemotherapy was initiated with two cycles of topotecan and carboplatin window treatment, followed by vincristine, actinomycin‐D, ifosfamide, adriamycin ± topotecan/carboplatin (VAIA/TC) in case of tumor response or carboplatin, epirubicin, vincristine, actinomycin‐D, ifosfamide, and etoposide (CEVAIE) chemotherapy in case of nonresponse.^11^ Treatment was continued with 6 months of oral trofosfamide, idarubicine, and etoposide (O‐TIE) maintenance. Patients not eligible for randomization were documented in CWS‐DOK‐IV 2004 and received CEVAIE chemotherapy, O‐TIE maintenance, and local treatment as described in the CWS‐IV 2002 protocol.^11^ In patients registered in SoTiSaR, treatment followed CWS Guidance, which proposed nine cycles CEVAIE chemotherapy and 6 months O‐TIE maintenance for all patients with metastatic disease.^14^

Local treatment was recommended after the fourth chemotherapy cycle in CWS‐2002‐IV or CWS Guidance^11^, ^14^ and after the sixth cycle in MTS 2008.^8^ In all protocols, surgical resection of the PT if feasible to achieve complete resection was uniformly recommended. Metastasectomy was specifically recommended in the CWS protocols, if feasible. In all protocols, radiotherapy to the PT and locoregional lymph node metastases was recommended, with radiotherapy to all distant metastatic sites if feasible. Radiation doses were adapted to histology, the type of resection (R0, R1, or R2) and response to chemotherapy ranging from 32 to 44.8 Gy hyperfractionated‐accelerated (CWS‐IV 2002), or 36–55.8 Gy conventionally fractionated (EpSSG MTS 2008, CWS Guidance). In MTS 2008, irradiation of the whole lung (WLI) with 15 Gy in 10 fractions starting concomitantly with the seventh cycle of chemotherapy was proposed for all patients with lung metastases.^8^, ^12^ In CWS‐IV 2002 and CWS Guidance, no clear statement on WLI was implemented.

### Data collection and evaluation

Treatment according to both trials and CWS Guidance was performed per requirements of the declaration of Helsinki and in accordance with the approval of the respective ethical committee. Written informed consent for data collection from the patient or from their legal guardians, or both, was required for all patients enrolled in the trials EpSSG MTS 2008, CWS‐IV 2002, or the registry SoTiSaR.

### Statistical methods

Statistical analyses were performed using R 4.2.3 (Vienna, Austria).^19^ Event‐free survival (EFS), lung–relapse‐free survival (lung‐RFS), and overall survival (OS) were calculated using the Kaplan–Meier estimator,^20^ and with confidence intervals (CI) stated at the 95% level. For OS, the time from diagnosis to death was calculated. For EFS, the time from diagnosis to progression, relapse after CR, diagnosis of secondary malignancy, or death was calculated. For lung‐RFS, time from diagnosis to relapsed or progressive disease affecting the lung was calculated. If there was no event, survival data were censored at last follow‐up, including patients lost to follow‐up which were censored at the time of the last known follow‐up. For comparison of EFS, lung‐RFS or OS levels across potential risk groups, the log‐rank test was used. Landmark analysis was implemented to adjust for the immortal time bias in retrospective analyses^21^ using a landmark time of 221 days (nine cycles of chemotherapy every 21 days plus 1‐month grace period^6^): progressive disease before the landmark time was excluded in analyses of treatment variables and delayed lung irradiation after the landmark time was defined as no irradiation.^12^ For exploratory subgroup analysis, univariable Cox proportional hazard models were implemented as previously described.^13^ Multivariable Cox proportional hazards regression model was developed to simultaneously assess the effect of prognostic factors on EFS and OS, with all variables not equally distributed between the three cohorts “CWS no lung RT,” “EpSSG no lung RT,” and “WLI+” being selected for the multivariable analysis. The proportional hazards (pH) assumption was examined by visually assessing Schoenfeld residuals and for variables violating the pH assumption, a continuous function to correct for time‐varying coefficients was implemented.^22^ All statistical tests were conducted at α = .05. This research is of exploratory nature and *p* values were not adjusted for multiple testing.

## RESULTS

### Patient characteristics and demography

**FIGURE 1:**
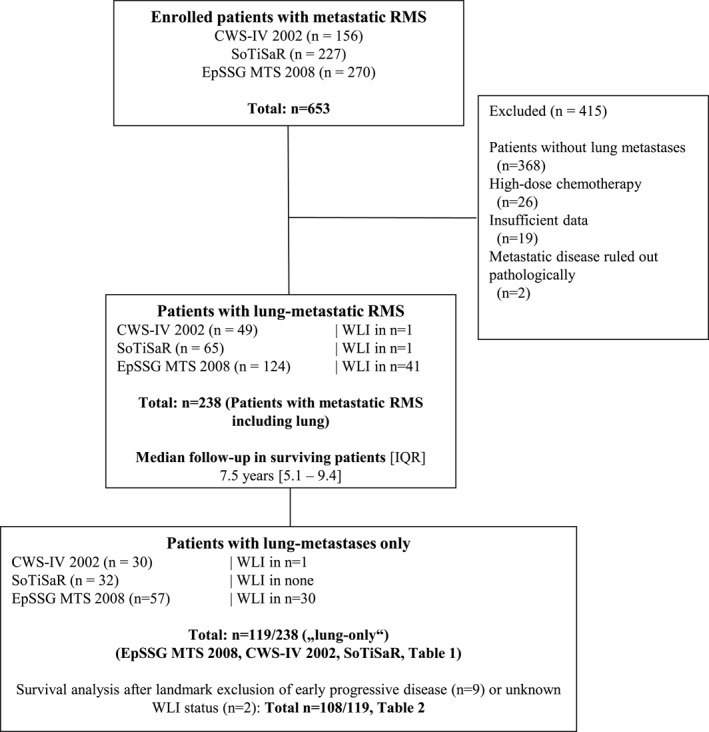
Consolidated Standards of Reporting Trials diagram depicting the selection of patients eligible for this analysis (*n* = 238). CWS, Cooperative Weichteilsarkom Studiengruppe; EpSSG, European pediatric Soft Tissue Sarcoma Study Group; MTS, metastatic tumors/sarcoma; RMS, rhabdomyosarcoma; SoTiSaR, Soft Tissue Sarcoma Registry; WLI, whole lung irradiation.

Patients with metastatic RMS were registered in CWS 2002‐IV (*n* = 156), the SoTiSaR registry (*n* = 227), and EpSSG MTS 2008 (*n* = 270). After the exclusion of patients without lung‐metastases (*n* = 368), receiving high‐dose chemotherapy or allogeneic stem cell transplantation (*n* = 26), insufficient data (*n* = 19), and two patients where lung metastases were not confirmed in the pathological evaluation, a total of *n* = 238 patients registered in EpSSG MTS 2008, CWS 2002‐IV, or the SoTiSaR registry were eligible for this analysis (Figure 1).

Exactly 50% (*n* = 119/238) of all patients had metastases confined to the lungs (“lung‐only”) and 50% were affected by lung metastases and other distant metastatic lesions (Table S1). Patients with lung‐met only disease were significantly younger with a median age of 6.6 years (standard deviation = ±4.7) compared to 11.5 years (standard deviation = ±5.5) in patients with lung and other distant metastatic lesions (*p* < .001); they were mainly diagnosed with ERMS (78% vs. 43%, *p* < .001), presented less nodal infiltration (35% vs. 60%, *p* < .001), more solitary lung nodules (23% vs. 13%, *p* = .046), and fewer Oberlin risk factors (Table S1). Biopsies of lung metastases were performed in *n* = 36/119 lung‐only RMS patients (30%), all other patients were classified as lung‐metastatic based on imaging.

### Treatment of lung‐only metastatic RMS

Regarding patients with lung‐only metastases (*n* = 119), only a small subgroup underwent upfront resection of the primary tumor (*n* = 12/118, unknown in *n* = 1, 10%) or upfront lung metastasectomy (*n* = 3/106, unknown in *n* = 13, 3%). Chemotherapy was administered according to protocol: IVADo (*n* = 57/119, 48%), VAIA/TC (*n* = 20/119, 19%), CEVAIE (*n* = 40/119, 33%), or other regimens in *n* = 2. Response of the primary tumor (CR + PR) after three cycles was observed in 91% of evaluable patients (*n* = 99/109, SD in *n* = 9, PD in *n* = 1). Two patients suffered from PD before response evaluation. Response evaluation of lung metastases revealed CR in 38% and non‐CR in 62% of 111 evaluable patients (no patient with PD). Comparing the different chemotherapy regimens, there was no difference concerning response rates of the primary tumor (*p* = .2) or lung metastases (*p* > .9).

Following chemotherapy, the primary tumor was resected in 79 patients and metastasectomy was performed in 14 (*n* = 6 in CWS‐IV 2002, *n* = 5 in SoTiSaR, and *n* = 3 in MTS 2008). The majority of patients underwent pre‐ or postoperative irradiation of the primary tumor (*n* = 94/116, unknown in *n* = 3, 81%) with a median dose of 50 Gy (range, 28–70 Gy). Lung irradiation was performed in 55% of patients in MTS 2008 within the landmark time (*n* = 31/57, one patient reclassified as “no WLI” due to delayed RT after 221 days after start of chemotherapy, unknown in *n* = 2), one patient in CWS‐IV 2002, and was never performed for patients included into the SoTiSaR registry. Mean dose was 15 Gy (14–15 Gy in 14 of 18 evaluable patients, 12 Gy in three patients, 18 Gy in one patient, and unknown dose in 13 patients). Two patients received a combination of WLI and pulmonary metastasectomy.

Maintenance treatment was administered with CYC/VNB in 42 patients, O‐TIE in 38 patients, and CYC/vinblastine in seven patients, the latter based on individual clinical decisions. Complete remission at the end of intensive treatment was achieved in 81 of 112 patients (72%, unknown in *n* = 7).

### Prognostic factors and outcome of lung‐only metastatic RMS

**FIGURE 2:**
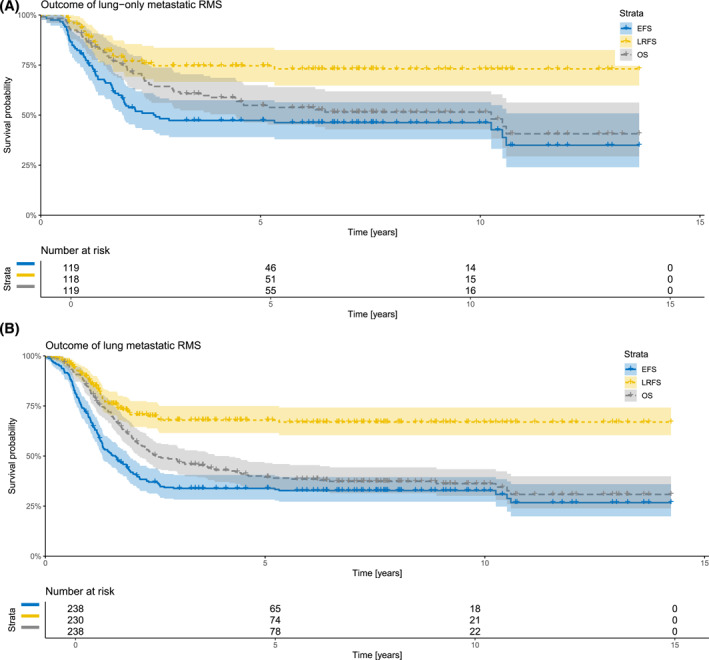
(A) Event‐free, lung–relapse‐free, and overall survival for lung‐only metastatic disease (*n* = 119). (B) Event‐free, lung–relapse‐free, and overall survival for patients with metastatic rhabdomyosarcoma including the lung (*n* = 238). EFS, event free survival; LRSF, lung relapse‐free survival; OS, overall survival; RMS, rhabdomyosarcoma.

The overall 5‐year EFS, lung‐RFS, and OS in patients with lung only metastatic RMS were 47% (95% CI, 39–57), 75% (95% CI, 67–84), and 55% (95% CI, 46–65), respectively (Figure 2A). Patients treated within EpSSG MTS 2008, CWS‐IV 2002, or registered in SoTiSaR had comparable 5‐year EFS (*p* = .4), lung‐RFS (*p* = .8), and OS (*p* = .4). Neither the Oberlin score nor histology, number of lung metastases, primary tumor size, or nodal status was predictive for patient outcomes (Table 1). Younger patients <10 years tended to demonstrate a higher 5‐year lung‐RFS of 80% (95% CI, 72–90) compared to 60% (95% CI, 44–82) in patients ≥10 years, but the difference did not reach statistical significance (*p* = .069). T2 status was associated with significantly improved lung‐RFS in the univariable analysis (*p* = .039), but this was not confirmed in a multivariable model (Figure S1).

**TABLE 1:** Univariate analysis of disease characteristics in *n* = 119 patients with lung‐only metastatic rhabdomyosarcoma.

Characteristic	*n* = 119, (in % of *n*)	5‐year EFS_YR (%; 95 CI)	*p*‐value (log rank test)	5‐year LRFS_YR (%; 95 CI)	*p*‐value (log rank test)	5‐year OS_YR (%; 95 CI)	*p*‐value (log rank test)
Cohort			.4		.8		.4
CWS‐IV‐2002	30 (25)	50% (35%, 72%)		78% (64%, 95%)		56% (41%, 77%)	
CWS‐SOTISAR	32 (27)	56% (41%, 78%)		77% (62%, 95%)		64% (48%, 85%)	
MTS2008	57 (48)	42% (31%, 57%)		72% (61%, 86%)		50% (39%, 65%)	
Sex			.3		.2		.4
Female	52 (44)	55% (42%, 70%)		82% (71%, 94%)		57% (44%, 73%)	
Male	67 (56)	42% (31%, 56%)		69% (58%, 82%)		54% (43%, 67%)	
Age, years			>.9		.069		.5
<10	88 (74)	49% (39%, 61%)		80% (72%, 90%)		57% (47%, 69%)	
≥10	31 (26)	43% (28%, 65%)		60% (44%, 82%)		48% (33%, 71%)	
Diagnosis			.8		>.9		>.9
RMA	21 (18)	40% (24%, 69%)		72% (55%, 96%)		50% (33%, 78%)	
RME	93 (78)	48% (39%, 60%)		75% (66%, 85%)		56% (47%, 68%)	
RMS	5 (4.2)	60% (29%, 100%)		80% (52%, 100%)		38% (8.4%, 100%)	
T size			.5		.2		.4
≤5 cm	25 (21)	52% (36%, 76%)		64% (47%, 88%)		60% (43%, 83%)	
>5 cm	92 (79)	45% (36%, 57%)		77% (68%, 87%)		53% (43%, 64%)	
T status			.2		.039		.4
T1	23 (20)	33% (19%, 60%)		57% (39%, 83%)		46% (29%, 72%)	
T2	92 (80)	51% (41%, 62%)		78% (70%, 88%)		57% (48%, 69%)	
N status			.4		.6		.2
N0	71 (65)	51% (41%, 65%)		75% (65%, 86%)		60% (49%, 72%)	
N1	38 (35)	42% (29%, 62%)		72% (58%, 90%)		47% (33%, 68%)	
Lung nodules			.8		.078		.6
Multiple	82 (77)	46% (36%, 58%)		69% (59%, 81%)		52% (42%, 65%)	
Solitary	25 (23)	43% (27%, 68%)		89% (76%, 100%)		54% (38%, 79%)	
Oberlin risk score			.4		.2		.13
0	56 (47)	56% (44%, 71%)		85% (76%, 96%)		66% (54%, 80%)	
1	54 (45)	39% (28%, 55%)		66% (54%, 81%)		46% (34%, 62%)	
2	9 (7.6)	44% (21%, 92%)		62% (37%, 100%)		42% (18%, 94%)	

Abbreviations: CI, confidence interval; EFS, event‐free survival; OS, overall survival; RFS, relapse‐free survival; RMA, alveolar rhabdomyosarcoma, RME, embryonal rhabdomyosarcoma, RMS, rhabdomyosarcoma, T size, tumor size; T status, Tumor status; N status, lymph node status; SoTISaR, Soft Tissue Sarcoma Registry.

**FIGURE 3:**
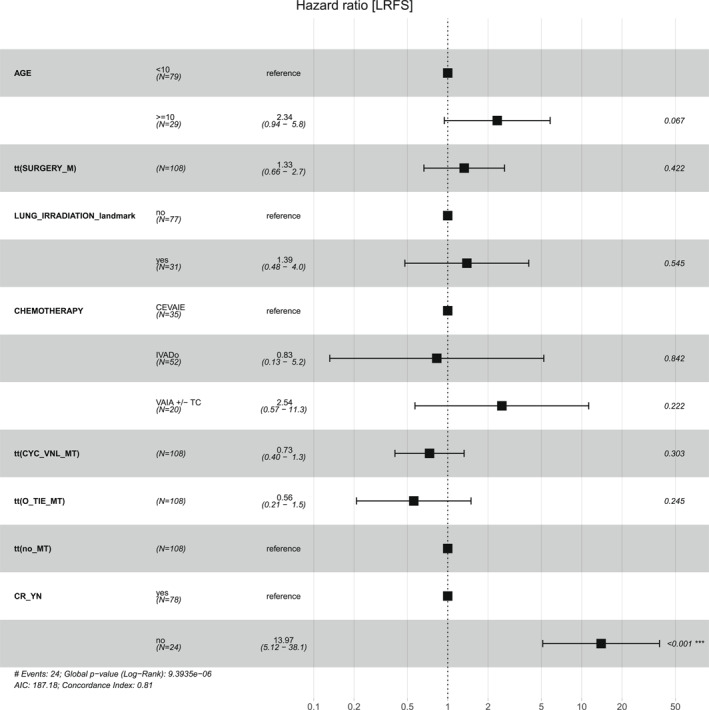
Multivariable Cox regression analysis evaluating the impact of the variables not equally distributed between the three cohorts “EpSSG no WLI,” “CWS no WLI,” and “WLI+” on lung‐RFS: age, surgery of metastases, lung irradiation, chemotherapy, maintenance chemotherapy with cyclophosphamide/vinorelbine, oral trofosfamide, idarubicin, and etoposide, or no maintenance and complete remission at the end of treatment. For variables violating the proportional hazards assumption, a continuous function to correct for time‐varying coefficients was implemented, these variables were marked with “tt.” CEVAIE, carboplatin, epirubicin, vincristine, actinomycin‐D, ifosfamide, etoposide; CR, complete remission; CWS, Cooperative Weichteilsarkom Studiengruppe; CYC, cyclophosphamide; EpSSG, European pediatric Soft Tissue Sarcoma Study Group; lung‐RFS, lung relapse‐free survival; IVADo, ifosfamide, vincristine, actinomycin‐D, doxorubicin; MT, maintenance treatment; N, no; O‐TIE, oral trofosfamide, idatubicine, etoposide; TC, topotecan, carboplatin; VAIA, vincristine, actinomycin‐D, ifosfamide, adriamycin; VNL, vinorelbine; WLI, whole lung irradiation; Y, yes.

For analysis of WLI, we excluded nine patients with progression before day 221 (landmark analysis) and two with missing RT data. Lung irradiation did not significantly improve 5‐year EFS (55% vs. 49%, *p* = .6) or lung‐RFS (76% vs. 74%, *p* = .6) (Table 2) in the univariable analyses. In contrast, CR of lung metastases after the initial three cycles of chemotherapy was associated with a nearly significant improvement of 5‐year EFS (61% [95% CI, 47–79] vs. 45% [95% CI, 34–59] in non‐CR patients, *p* = .051) and a highly significant improvement of 5‐year OS (*p* = .009), but not of lung‐RFS (*p* = .3). Lung irradiation or pulmonary metastasectomy did not improve lung‐RFS in the multivariate analysis, in contrast to complete remission at the end of treatment (Figure 3).

**TABLE 2:** Univariable analysis of treatment in patients with lung‐only metastatic rhabdomyosarcoma after exclusion of progression before 221 days (*n* = 9) or unknown lung RT status (*n* = 2).

Characteristic	*n* = 108 (in % of n)	5‐year EFS_YR (%; 95 CI)	*p*‐value (log rank test)	5‐year LRFS_YR (%; 95 CI)	*p*‐value (log rank test)	5‐year OS_YR (%; 95 CI)	*p*‐value (log rank test)
Subgroup (WLI)			.041		.3		.035
CWS no lung RT	55 (51)	57% (45%, 72%)		78% (67%, 91%)		65% (53%, 79%)	
EPSSG no lung RT	22 (20)	32% (17%, 59%)		65% (47%, 90%)		41% (25%, 68%)	
WLI+	31 (29)	55% (40%, 75%)		76% (62%, 93%)		61% (45%, 81%)	
Chemotherapy			.13		.3		.12
CEVAIE	35 (33)	67% (52%, 85%)		84% (72%, 98%)		75% (61%, 92%)	
IVADo	52 (49)	46% (34%, 62%)		73% (62%, 87%)		53% (41%, 69%)	
VAIA +/– TC	20 (19)	35% (19%, 64%)		63% (44%, 89%)		44% (27%, 73%)	
Other	1						
Response of primary tumor (weeks 7–10)			.6		.4		.5
CR	6 (5.9)	33% (11%, 100%)		83% (58%, 100%)		50% (22%, 100%)	
PR	86 (85)	52% (43%, 64%)		77% (68%, 87%)		59% (49%, 71%)	
SD	9 (8.9)	44% (21%, 92%)		56% (31%, 100%)		44% (21%, 92%)	
Not assessable	7						
Response of lung metastases (weeks 7–10)			.051		.3		.009
CR	39 (38)	61% (47%, 79%)		81% (69%, 95%)		71% (58%, 87%)	
Non‐CR	63 (62)	45% (34%, 59%)		70% (59%, 83%)		50% (38%, 64%)	
Not assessable	6						
Best surgery of PT			.7		>.9		.6
R0	21 (52)	62% (44%, 87%)		76% (60%, 97%)		71% (54%, 94%)	
R1	10 (25)	60% (36%, 100%)		80% (59%, 100%)		70% (47%, 100%)	
R2	9 (22)	44% (21%, 92%)		78% (55%, 100%)		53% (28%, 100%)	
Unknown	7						
Surgery of PT at any time			.9		.5		>.9
Yes	46 (43)	54% (41%, 71%)		79% (67%, 92%)		62% (50%, 78%)	
No	61 (57)	49% (38%, 64%)		71% (60%, 84%)		54% (43%, 69%)	
Unknown	1						
Pulmonary metastasectomy			.4		.5		.2
Yes	16 (15)	43% (24%, 76%)		64% (43%, 96%)		43% (23%, 80%)	
No	92 (85)	52% (43%, 64%)		76% (68%, 86%)		61% (51%, 72%)	
RT of primary tumor			.5		.4		.6
Yes	91 (85)	53% (43%, 64%)		76% (67%, 86%)		59% (49%, 71%)	
No	16 (15)	44% (25%, 76%)		66% (46%, 95%)		56% (37%, 87%)	
Unknown	1						
Lung irradiation^a^			.6		.6		.7
Yes	31 (29)	55% (40%, 75%)		76% (62%, 93%)		61% (45%, 81%)	
No	77 (71)	49% (39%, 62%)		74% (65%, 85%)		57% (47%, 70%)	
Maintenance chemotherapy			.4		.5		.3
Yes (CYC/VBL)	7 (6.7)	71% (45%, 100%)		71% (45%, 100%)		71% (45%, 100%)	
Yes (CYC/VNL)	42 (40)	50% (37%, 68%)		79% (68%, 93%)		57% (43%, 74%)	
Yes (O‐TIE)	36 (35)	54% (40%, 74%)		76% (63%, 92%)		63% (49%, 82%)	
No	19 (18)	46% (28%, 76%)		63% (44%, 92%)		51% (32%, 80%)	
Unknown	4						
Complete remission at the end of treatment			<.001		<.001		<.001
Yes	78 (76)	65% (55%, 76%)		87% (79%, 95%)		73% (64%, 84%)	
No	24 (24)	4.2% (0.6%, 28%)		17% (5.0%, 56%)		8.3% (2.2%, 31%)	
Unknown	6						

Abbreviations: CEVAIE, cyclophosphamide, epirubicin, vincristine, acto‐D, ifosfamide, etoposide; CI, confidence interval; CR, complete response; CYC/VBL, cyclophosphamide/vinblastine; CYC/VNB, cyclophosphamide/vinorelbine; EFS, event‐free survival; EpSSG, European pediatric Soft Tissue Sarcoma Study Group; IVADo, ifosfamide, vincristine, acto‐D, doxorubicin; OS, overall survival; O‐TIE, oral trofosfamide, idarubicine, etoposide; PR, partial response; PT, primary tumor; R0, microscopically complete resection; R1, microscopically incomplete resection; R2, macroscopically incomplete resection; RT, radiotherapy, SD, stable disease; TC, topotecan, carboplatin; VAIA, vincristine, actionymcin‐D, ifosfamide, adriamycin; VNL, vinorelbine; WLI, whole lung irradiation.

^a^ Delayed lung irradiation after day 221 classified as no irradiation (landmark analysis).

**FIGURE 4:**
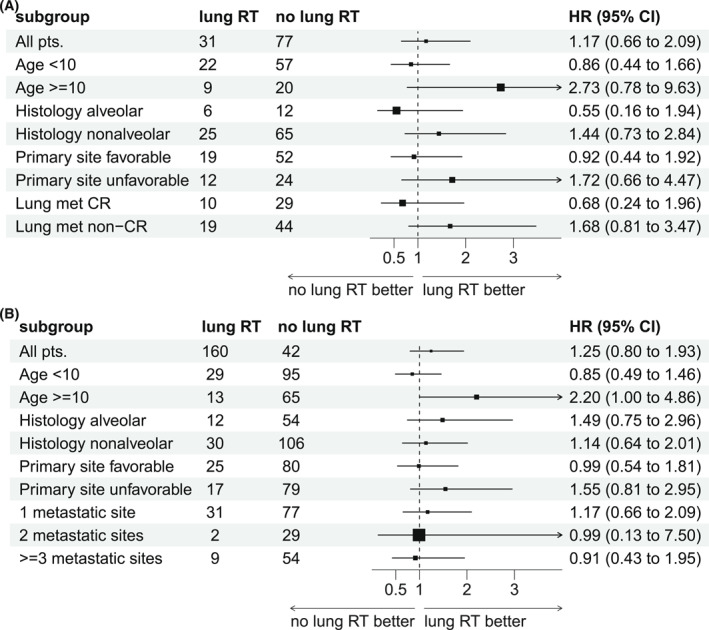
(A) Univariable Cox regression depicting the impact of lung irradiation on event‐free survival in different subgroups of lung‐only metastatic RMS patients using in a forest plot. (B) Univariable Cox regression depicting the impact of lung irradiation on event‐free survival in different subgroups of the whole cohort of lung‐metastatic RMS patients. pts, patients; RMS, rhabdomyosarcoma; RT, radiotherapy.

For the purpose of this analysis, three cohorts were defined: patients in the CWS or the EpSSG cohort not undergoing WLI (“CWS no WLI” or “EpSSG no WLI”, respectively) and patients from both study groups undergoing WLI (“WLI+”). Analyzing the different patient cohorts, we found a significantly inferior 5‐year EFS in the EpSSG “no WLI” group with 32% (95% CI, 17–59) when compared to the CWS “no WLI” cohort with 57% (95% CI, 45–72) or the combined “WLI+” cohort with 55% (95% CI, 40–75; *p* = .041). Regarding 5‐year lung‐RFS, no significant difference between the three cohorts could be demonstrated (Table 2). Age, chemotherapy regimen, maintenance treatment regimen, lung metastasectomy, and lung irradiation (Table S2) were variables unequally distributed between the three cohorts. Univariable analyses revealed that none of these variables were significantly correlated with EFS, lung‐RFS, or OS (Table 2). A detailed investigation regarding the effect of lung irradiation in different subgroups by using univariable Cox regression models indicated that no subgroup benefited (Figure 4A).

### Analysis on the impact of lung irradiation in patients with RMS lung‐only and lung plus other metastases

The outcome of the whole cohort (patients with lung metastases only plus patients with lung metastases and other metastatic lesions, total *n* = 238) (Figure 2B) was significantly worse compared to lung only metastatic patients with a 5‐year EFS, lung‐RFS, and OS of 21% (95% CI, 14–29; *p* < .001), 60% (95% CI, 50–72; *p* = .03), and 24% (95% CI, 17–34; *p* < .001), respectively. After landmark exclusion of early PD (*n* = 36), the effect of lung irradiation was evaluated using univariable analyses and revealed no benefit on 3‐year EFS with 42% (95% CI, 33–55; *n* = 42) in irradiated patients compared to 38% (95% CI, 31–46; *n* = 160) in nonirradiated patients (*p* = .3), and no effect on lung‐RFS (*p* = .5). Subgroup analyses on the effect of lung irradiation including the same variables as described in a comparable patient cohort^13^ using univariable Cox regression models revealed a significant influence on EFS in older (≥10 years) patients only (hazard ratio, 2.2; 95% CI, 1.0–4.86) (Figure 4B).

## DISCUSSION

With this pooled European retrospective analysis, we present the largest data set on patients with lung‐metastatic rhabdomyosarcoma published to date. The analysis primarily focuses on the subgroup of patients with lung‐only metastatic RMS to be able to investigate the effects of whole lung irradiation in a homogeneous cohort. The reported 5‐year EFS and OS of 47% and 55% in patients with lung‐only metastatic RMS was significantly better than the 21% and 24% in the cohort of lung‐metastatic patients with and without other metastatic sites. This also aligns with recently published data.^3^, ^13^ Initial disease characteristics could not be used to refine risk stratification for patients with lung‐only metastatic RMS, because none of these variables had a significant impact on EFS, lung‐RFS, or OS, being consistent with reports from smaller cohorts.^3^, ^10^, ^12^

We were able to show that outcomes did not significantly differ between patients in EpSSG MTS 2008, CWS‐IV 2002, and SoTiSaR, although different chemotherapy regimens were used for systemic treatment, similar to recent analyses.^14^, ^23^, ^24^ Local treatment of the primary tumor with resection and/or RT did not differ between the different trials or the SoTiSaR registry, which can be explained by the fact that the treatment protocols were very similar. However, as treatment protocols concerning the treatment of metastases varied and as EpSSG protocol explicitly recommended WLI, we were able to investigate the potential benefit of lung irradiation.

We could not demonstrate a benefit of WLI in patients with lung‐only metastatic disease confirming data from a smaller COG cohort.^3^

The “EpSSG no WLI” group showed poorer outcomes compared to nonirradiated CWS patients as well as to irradiated patients. Because the comparison of three different cohorts treated on the basis of different protocols is prone to systematic bias, we conducted detailed analyses to identify potential confounders. There were more patients older than 10 years (leading to higher Oberlin scores) and more patients with nonremission in the “EpSSG no WLI” group compared to the other patient cohorts. Those with nonremission at the end of treatment had a significantly worse outcome in the univariable as well as in the multivariable analyses, whereas age, WLI, or pulmonary metastasectomy were not significant factors. Nonremission at the end of treatment may indicate a more aggressive biologic behavior of these tumors, diminishing patient survival rates regardless of WLI. Early CR of lung metastases was associated with improved overall survival as previously described^10^ and was correlated with a 3‐year lung‐RFS of 81%, showing that lung relapse was very rare in this subgroup. Notably, lung‐RFS of these patients was significantly higher than both EFS and OS in all analyses and was not influenced by WLI, hence the efficacy of lung irradiation in patients with lung‐only metastatic RMS cannot be proven by these data. The additional analysis of lung‐RFS as well as the identification of a potential selection bias between the “EpSSG no WLI” group compared to the “CWS no WLI” and “WLI+” subgroups might explain why the benefit of WLI in lung‐only RMS could not be proven, contrasting the previously published analysis of the EpSSG patients.^12^

It might be an issue that a dose of 15 Gy, which is significantly lower than the dose typically recommended for radiotherapy of RMS,^25^ is not sufficient to prevent lung relapse in these patients. However, there are limitations that might introduce bias in the post hoc analysis of WLI: the overall number of lung‐irradiated patients was small and lower than of nonirradiated patients, the assignment to WLI in EpSSG MTS 2008 was not randomized, and the reasons for nonirradiation are unknown. In addition, although a radiation dose of 15 Gy was administered to approximately 80% of the evaluable patients, there were patients only receiving 12 Gy of WLI, as well as patients for whom no dose was documented. Given the small numbers of patients with deviating dose prescriptions, a detailed investigation of dose‐dependent effects was not possible, and a potential negative impact of dose deviations on the efficacy of WLI cannot be ruled out.

To compare our results with the very recently published COG analysis demonstrating efficacy of WLI in RMS patients with distant metastases in the lung and other organ sites,^13^ we performed an analysis including all patients (lung metastases only and lung plus other distant metastases). Here, we could confirm a significant impact of WLI in patients ≥10 years in our data. However, WLI did not improve EFS nor OS of the whole group of lung‐irradiated patients, contrasting with the published results mentioned above. The difference concerning the overall efficacy of WLI in disseminated RMS may first be attributed to the older age of patients in the COG cohort composed of high‐risk studies^13^ as in COG, patients with <10 years and ERMS are usually treated in intermediate‐risk protocols not always including WLI.^26^ Second, there were more patients with high‐risk metastatic RMS (≥2 Oberlin risk factors) included in the COG cohort, possibly reflecting a different biologic aggressiveness of disease. The earlier landmark time set (12 weeks in D9803, 15 weeks in D9802, and 20 weeks in ARST0431/08P1^13^ compared to 31 weeks in our analysis) and the inclusion of patients with delayed RT (>90 days after primary RT in 28% of patients)^13^ in the irradiated COG cohort is allowing more survivorship bias.^21^ Last, it is not clear if the irradiated COG patients also had RT applied to other metastatic sites—if they did, it is possible that the improved survival is related to radical irradiation of all sites^6^ rather than to WLI.

Importantly, the potential benefit of WLI has to be weighed against the potential long‐term sequelae in pediatric age. In longitudinal analyses in pediatric patients with solid malignancies including RMS, the proportion of patients with pathological spirometry and reduced total lung capacity after WLI was above 50% after a median follow‐up of 9.7 years, with a subsequent decline over the observational period.^27^, ^28^ Growth disruption of the thoracic bones as well as the female breast was frequent.^29^ The rate of secondary malignancies is doubled through irradiation as part of the sarcoma treatment.^30^

In conclusion, our analysis failed to show a benefit of WLI in lung‐metastatic RMS patients, except for the subgroup of patients older than 10 years. A randomized trial on WLI versus no WLI is needed to finally clarify its role similar to Ewing sarcoma patients, but recruiting enough patients will probably need a vast amount of time.^31^ Additionally, to further investigate these points, a retrospective analysis on a larger cohort of patients including COG patients within the INSTRuCT database^32^ is planned.

## AUTHOR CONTRIBUTIONS

**Amadeus T. Heinz**: Conceptualization; visualization; validation; methodology; investigation; formal analysis; data curation; writing—original draft; writing—review and editing; software; project administration. **Philip Heesen**: Visualization; methodology; investigation; formal analysis; validation; writing—review and editing; software. **Johannes H. M. Merks**: Conceptualization; validation; data curation; funding acquisition; writing—review and editing; resources. **Véronique Minard‐Colin**: Conceptualization; validation; data curation; writing—review and editing; funding acquisition; resources. **Martin Ebinger**: Validation; writing—review and editing; resources. **Alison L. Cameron**: Validation and writing—review and editing. **Semi Harrabi**: Validation; writing—review and editing. **Henry Mandeville**: Validation; writing—review and editing. **Reineke A. Schoot**: Conceptualization; validation; data curation; writing—review and editing; resources; funding acquisition. **Jörg Fuchs**: Validation; writing—review and editing. **Gabriela Guillen**: Validation; writing—review and editing. **Ewa Koscielniak**: Conceptualization; validation; data curation; writing—review and editing; funding acquisition; resources. **Julia C. Chisholm**: Conceptualization; validation; funding acquisition; writing—review and editing; data curation; resources. **Monika Sparber‐Sauer**: Conceptualization; methodology; data curation; validation; writing—review and editing; project administration; supervision; resources. **Gianni Bisogno**: Conceptualization; validation; methodology; data curation; writing—review and editing; funding acquisition; project administration; supervision; resources.

## CONFLICT OF INTEREST STATEMENT

Julia C. Chisholm reports participation on a data and safety monitoring board for the Children’s Oncology Group; fees for professional activities from the National Cancer Institute; and grant and/or contract funding from Bayer. Martin Ebinger reports grant and/or contract funding from Amgen. Henry Mandeville reports consulting fees from the Institute of Cancer Research and the University College London Hospitals NHS Foundation Trust. Johannes H. M. Merks reports consulting fees from Bayer, GlaxoSmithKline, and Merck. Veronique Minard‐Colin reports grant and/or contract funding from F. Hoffmann‐La Roche. Monika Sparber‐Sauer has acted as consultant and/or advisory board member for Roche, Bayer and Swedish Orphan Biovitrum (hemophilia); and is partially supported by Bayer (investigation supported research) for an independent project on NTRK‐positive tumors. The other authors declare no conflicts of interest.

## Supporting information

Supporting Information S1

Figure S1

Table S1

Table S2

## Data Availability

The data that support the findings of this study are available from the corresponding author on reasonable request.
