# Supplementary material for: The impact of whole lung irradiation in lung metastatic rhabdomyosarcoma: A pooled analysis of two European trials and one European registry
Source: Cancer. 2026 Jul 23;132(15):e70530. doi: 10.1002/cncr.70530 (PMC13395302; doi:10.1002/cncr.70530)
Supplement: Supplementary file 1 — Supporting Information S1 [file CNCR-132-e70530-s001.docx]

SUPPLEMENTAL FIGURE 1: Multivariable Cox regression analysis evaluating the prognostic factors Oberlin risk score, histology, tumor size, N status and T status in patients with lung-only metastatic RMS (n=11)
