# Supplementary figures and images for: The impact of whole lung irradiation in lung metastatic rhabdomyosarcoma: A pooled analysis of two European trials and one European registry
Source: Cancer. 2026 Jul 23;132(15):e70530. doi: 10.1002/cncr.70530 (PMC13395302; doi:10.1002/cncr.70530)

# Hazard ratio [LRFS]

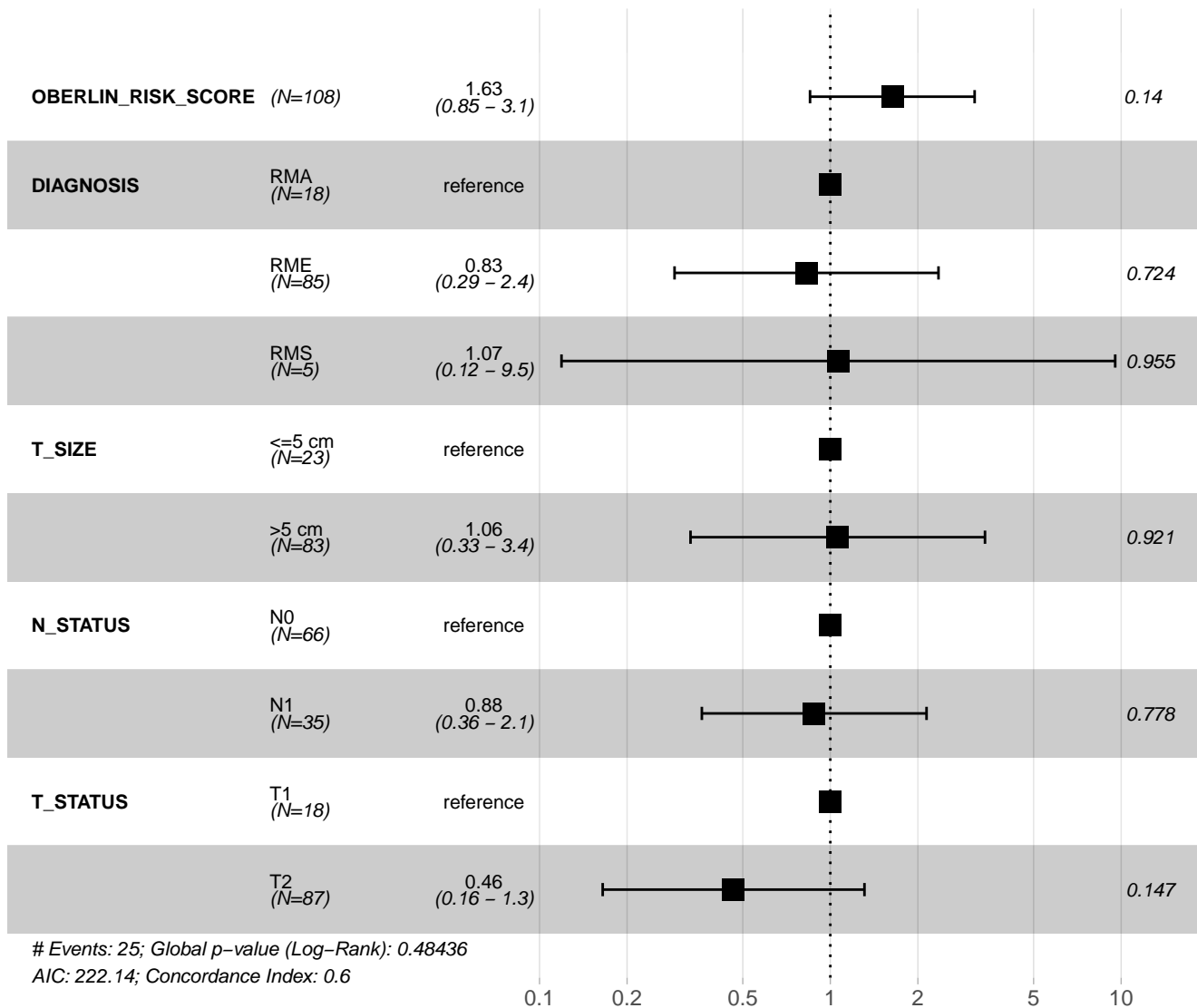

Supplement: Supplementary file 2 — Figure S1 [file CNCR-132-e70530-s003.pdf]
