# Supplementary material for: The impact of whole lung irradiation in lung metastatic rhabdomyosarcoma: A pooled analysis of two European trials and one European registry
Source: Cancer. 2026 Jul 23;132(15):e70530. doi: 10.1002/cncr.70530 (PMC13395302; doi:10.1002/cncr.70530)
Supplement: Supplementary file 3 — Table S1 [file CNCR-132-e70530-s004.docx]

**Supplementary Table 1**: Differences between lung-only metastatic RMS and RMS with lung + other metastatic sites (n=238 total)

| Characteristic | Lung only metastases,  N = 119 | Lung and other metastases,  N = 119 | p-value2 |
| --- | --- | --- | --- |
| COHORT |  |  | 0.2 |
| CWS-IV-2002 | 30 (25%) | 19 (16%) |  |
| CWS-SOTISAR | 32 (27%) | 33 (28%) |  |
| MTS2008 | 57 (48%) | 67 (56%) |  |
| SUBGROUP |  |  | <0.001 |
| CWS no lung RT | 59 (50%) | 49 (41%) |  |
| EPSSG no lung RT | 29 (22%) | 53 (45%) |  |
| WLI+ (CWS and EpSSG) | 31 (27%) | 17 (14%) |  |
| GENDER |  |  | 0.7 |
| female | 52 (44%) | 49 (41%) |  |
| male | 67 (56%) | 70 (59%) |  |
| AGE |  |  | <0.001 |
| <10 years | 88 (74%) | 55 (46%) |  |
| >=10 years | 31 (26%) | 64 (54%) |  |
| DIAGNOSIS |  |  | <0.001 |
| ARMS | 21 (18%) | 60 (50%) |  |
| ERMS | 93 (78%) | 51 (43%) |  |
| RMS NOS | 5 (4.2%) | 8 (6.7%) |  |
| T_SIZE |  |  | 0.3 |
| <=5 cm | 25 (21%) | 18 (16%) |  |
| >5 cm | 92 (79%) | 92 (84%) |  |
| T_STATUS |  |  | 0.7 |
| T1 | 23 (20%) | 25 (22%) |  |
| T2 | 92 (80%) | 90 (78%) |  |
| N_STATUS |  |  | <0.001 |
| N0 | 71 (65%) | 42 (40%) |  |
| N1 | 38 (35%) | 64 (60%) |  |
| LUNG_NODULES | |  | 0.046 |
| solitary | 25 (23%) | 15 (13%) |  |
| multiple | 82 (77%) | 100 (87%) |  |
| OBERLIN_RISK_SCORE | |  | <0.001 |
| 0 | 56 (47%) | 4 (3.4%) |  |
| 1 | 54 (45%) | 22 (18%) |  |
| 2 | 9 (7.6%) | 29 (24%) |  |
| 3 | 0 (0%) | 42 (35%) |  |
| 4 | 0 (0%) | 22 (18%) |  |
| 1 n (%) |  |  |  |
| 2 Pearson’s Chi-squared test | | |  |
