# Supplementary material for: The impact of whole lung irradiation in lung metastatic rhabdomyosarcoma: A pooled analysis of two European trials and one European registry
Source: Cancer. 2026 Jul 23;132(15):e70530. doi: 10.1002/cncr.70530 (PMC13395302; doi:10.1002/cncr.70530)
Supplement: Supplementary file 4 — Table S2 [file CNCR-132-e70530-s002.docx]

Supplementary Table 2: Characteristics l**ung RT neg. vs. lung RT** **pos.** subgroups, **lung-met only RMS** after **landmark analysis** (n=108, exclusion of early progression before day 221 n=9, exclusion of lung RT n/a n=2)

| Characteristic | CWS no lung RT,  N = 55 | EPSSG no lung RT, N = 22 | Lung RT+,  N = 31 | p-value |
| --- | --- | --- | --- | --- |
| COHORT |  |  |  | **<0.001** |
| CWS-IV-2002 | 27 (49%) | 0 (0%) | 1 (3.2%) |  |
| CWS-SOTISAR | 28 (51%) | 0 (0%) | 0 (0%) |  |
| MTS2008 | 0 (0%) | 22 (100%) | 30 (97%) |  |
| GENDER |  |  |  | 0.8 |
| female | 25 (45%) | 8 (36%) | 13 (42%) |  |
| male | 30 (55%) | 14 (64%) | 18 (58%) |  |
| AGE |  |  |  | **0.01** |
| <10 | 46 (84%) | 11 (50%) | 22 (71%) |  |
| >=10 | 9 (16%) | 11 (50%) | 9 (29%) |  |
| DIAGNOSIS |  |  |  | 0.8 |
| RMA | 7 (13%) | 5 (23%) | 6 (19%) |  |
| RME | 45 (82%) | 16 (73%) | 24 (77%) |  |
| RMS | 3 (5.5%) | 1 (4.5%) | 1 (3.2%) |  |
| T_SIZE |  |  |  | 0.9 |
| <=5 cm | 13 (24%) | 4 (18%) | 6 (20%) |  |
| >5 cm | 41 (76%) | 18 (82%) | 24 (80%) |  |
| T_STATUS |  |  |  | 0.8 |
| T1 | 8 (15%) | 4 (18%) | 6 (19%) |  |
| T2 | 44 (85%) | 18 (82%) | 25 (81%) |  |
| N_STATUS |  |  |  | 0.2 |
| N0 | 28 (57%) | 17 (77%) | 21 (70%) |  |
| N1 | 21 (43%) | 5 (23%) | 9 (30%) |  |
| LUNG_NODULES | |  |  | >0.9 |
| multiple | 34 (79%) | 18 (82%) | 24 (77%) |  |
| solitary | 9 (21%) | 4 (18%) | 7 (23%) |  |
| OBERLIN_RISK_SCORE | |  |  | 0.12 |
| 0 | 32 (58%) | 6 (27%) | 13 (42%) |  |
| 1 | 20 (36%) | 13 (59%) | 15 (48%) |  |
| 2 | 3 (5.5%) | 3 (14%) | 3 (9.7%) |  |
| CHEMOTHERAPY | | |  | **<0.001** |
| CEVAIE | 35 (65%) | 0 (0%) | 0 (0%) |  |
| IVADo | 0 (0%) | 22 (100%) | 30 (97%) |  |
| VAIA+-TC | 19 (35%) | 0 (0%) | 1 (3%) |  |
| other | 1 | | |  |
| RESPONSE_PT (week 7-10) | |  |  | >0.9 |
| CR | 3 (6.0%) | 1 (4.5%) | 2 (6.9%) |  |
| PR | 43 (86%) | 19 (86%) | 24 (83%) |  |
| SD | 4 (8.0%) | 2 (9.1%) | 3 (10%) |  |
| RESPONSE_LUNG_MET (week 7-10) | | |  | 0.6 |
| CR | 19 (37%) | 10 (48%) | 10 (34%) |  |
| non-CR | 33 (63%) | 11 (52%) | 19 (66%) |  |
| BEST_SURGERY_PT | |  |  | >0.9 |
| R0 | 8 (53%) | 5 (45%) | 8 (57%) |  |
| R1 | 4 (27%) | 3 (27%) | 3 (21%) |  |
| R2 | 3 (20%) | 3 (27%) | 3 (21%) |  |
| SURGERY_PT_ANY_TIME | |  |  | 0.6 |
| no | 33 (61%) | 11 (50%) | 17 (55%) |  |
| yes | 21 (39%) | 11 (50%) | 14 (45%) |  |
| SURGERY lung metastases at any time | |  |  | **0.004** |
| no | 41 (75%) | 22 (100%) | 29 (94%) |  |
| yes | 14 (25%) | 0 (0%) | 2 (6.5%) |  |
| RT_PT |  |  |  | 0.3 |
| no | 10 (19%) | 4 (18%) | 2 (6.5%) |  |
| yes | 44 (81%) | 18 (82%) | 29 (94%) |  |
| MAINTENANCE_CHT | |  |  | **<0.001** |
| no | 9 (18%) | 6 (27%) | 4 (13%) |  |
| yes (CYC/VBL) | 6 (12%) | 0 (0%) | 1 (3.2%) |  |
| yes (CYC/VNL) | 0 (0%) | 16 (73%) | 26 (84%) |  |
| yes (O-TIE) | 36 (71%) | 0 (0%) | 0 (0%) |  |
| Complete remission end of treatment |  |  |  | **0.033** |
| no | 8 (16%) | 10 (45%) | 6 (19%) |  |
| yes | 41 (84%) | 12 (55%) | 25 (81%) |  |
| 1 n (%) |  |  |  |  |
